# CXCL12-stimulated lymphocytes produce secondary stimulants that affect the surrounding cell chemotaxis

**DOI:** 10.1016/j.bbrep.2021.101128

**Published:** 2021-09-07

**Authors:** Kaoru Kurowarabe, Masataka Endo, Daichi Kobayashi, Haruko Hayasaka

**Affiliations:** a Department of Science, Graduate School of Science and Engineering, Kindai University, 3-4-1, Kowakae, Higashiosaka, Osaka, 577-8502, Japan; b Laboratory of Immune Molecular Function, Faculty of Science and Engineering, Kindai University, 3-4-1, Kowakae, Higashiosaka, Osaka, 577-8502, Japan; c Department of Immunology, Niigata University Graduate School of Medical and Dental Sciences, 1-757 Asahimachi-dori, Chuo-ku, Niigata, 951-8510, Japan; d Research Institute for Science and Technology, Kindai University, 3-4-1, Kowakae, Higashiosaka, Osaka, 577-8502, Japan

**Keywords:** Lymphocyte, Chemokine, Neutrophil, Migration, Chemotaxis, CXCR4

## Abstract

Chemotactic factors locally secreted from tissues regulate leukocyte migration via cell membrane receptors that induce intracellular signals. It has been suggested that neutrophils stimulated by bacterial peptides secrete a secondary stimulant that enhances the chemotactic cell migration of the surrounding cells. This paracrine mechanism contributes to chemokine-dependent neutrophil migration, however, it has not yet been extensively studied in lymphocytes. In this study, we provide evidence that lymphocytes stimulated by the chemokine, CXCL12, affect the CXCR4-independent chemotactic response of the surrounding cells. We found that CXCR4-expressing lymphocytes or the conditioned medium from CXCL12-stimulated cells promoted CXCR4-deficient cell chemotaxis. In contrast, the conditioned medium from CXCL12-stimulated cells suppressed CCR7 ligand-dependent directionality and the cell migration speed of CXCR4-deficient cells. These results suggest that paracrine factors from CXCL12-stimulated cells navigate surrounding cells to CXCL12 by controlling the responsiveness to CCR7 ligand chemokines and CXCL12.

## Highlights

● CXCL12-stimulated lymphocytes affect the CXCR4-independent chemotactic response of the surrounding cells.
● The conditioned medium from CXCL12-stimulated cells promoted CXCR4-deficient cell chemotaxis, whereas it suppresses CCR7 ligand-dependent directionality and the cell migration speed.
● The CXCL12/CXCR4 axis causes the production of a signal-relay molecule that contributes to chemokine-dependent lymphocyte migration.

## Graphical abstract

**Figure:**
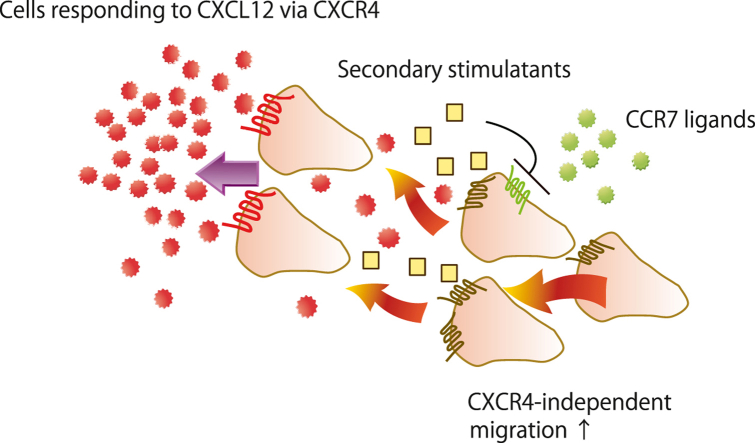

## 1 Introduction

The directed recruitment of cells by chemotactic factors is an important aspect of various biological processes, including wound healing, angiogenesis, the immune response, and cancer metastasis [1]. Chemokines control the recruitment and positioning of cells to the local tissue by inducing intracellular signaling via transmembrane G protein-coupled receptors (GPCRs) [2,3]. In lymphocyte migration, multiple chemokines, including CCL21/SLC, CCL19/ELC, CXCL12/stromal cell-derived factor 1 alpha, CXCL10/IP-10, and CXCL13/BLC, mediate chemokine gradient-dependent cell migration and contribute to efficient lymphocyte migration from the blood to the lymph nodes and Payer's patches [4]. Both CCL21 and CCL19 bind to a common receptor, CCR7, which is critical for T cell trafficking through high endothelial venules in the lymph nodes and Payer's patches [5,6]. CXCL12 is expressed in multiple tissues, including lymphoid tissues, the lungs, liver, and brain, and selectively binds to its receptors, CXCR4 and CXCR7, functioning as a chemoattractant and controlling efficient naïve lymphocyte migration in concert with CCR7 ligand chemokines [7,8]. CXCR4 is known as a major receptor in various biological processes, including leukocyte migration, maintenance of hematopoietic stem cell niches, tissue development, and cancer metastasis [[9], [10], [11]]. In contrast to CXCR4, CXCR7 only contributes to lymphocyte migration to a minor extent due to its low expression level on leukocyte cell surfaces under homeostatic conditions [12].

In recent years, it has been proposed that leukocyte migration is regulated by sequential waves of multiple chemoattractants, which propagate cell migration signals triggered by a primary stimulus to the surrounding cells. For example, neutrophils stimulated by formyl-Met-Leu-Phe (fMLP) extracellularly secrete a secondary chemoattractant, leukotriene B4 (LTB4), which further amplifies fMLP-mediated neutrophil migration [13]. Neutrophils also self-regulate their recruitment by the autocrine production of the chemokine, CXCL2, which further acts as a paracrine chemoattractant for other neutrophils [14]. Although the paracrine action of substances is a widespread concept in neutrophil recruitment to the site of inflammation, it is unknown whether a similar mechanism is involved in chemokine-dependent lymphocyte migration to secondary lymphoid tissues. In this study, we investigated whether CXCL12 stimulation induces the production of secondary substances from human T lymphocytes, which act as paracrine messengers to propagate chemokine signals to the surrounding cells.

## 2 Materials and methods

### 2.1 Cell culture and establishment of CXCR4-KO H9 cells

The human CD4^+^ T cell line, H9 (HTB-176), was obtained from the American Type Culture Collection and cultured in 10% (v/v) fetal calf serum (FCS; PAA Laboratories), 10 mM HEPES (Gibco), and 1 M sodium pyruvate in RPMI1640 medium (Sigma) containing 100 U/ml penicillin (Gibco), 100 μg/mL streptomycin (Gibco), 0.1 mM non-essential amino acids (Gibco), and 50 μM 2-mercaptoethanol (Sigma).

The CXCR4-knockout (KO) H9 cell line was established using the CRISPR-Cas9 system as follows. First, the double-stranded oligonucleotide corresponding to nucleotides 337–356 of the human CXCR4 gene (NM 0010084540.1) was subcloned to the plasmid vector GeneArt CRISPR (ThermoFisher Scientific) with orange fluorescent protein (OFP) and Cas9 nuclease genes; then the plasmid was gene-introduced into H9 cells using a Nucleofector (Lonza). The transfected cells were expanded and subjected to cell sorting using a FACS Aria II cell sorter (BD Bioscience). The purified OFP-positive H9 cells were subjected to limiting dilution in 96-well plates to generate a monoclonal CXCR4-KO cell line. The CXCR4 sequences contained in the guide RNA are described below.

CXCR4 target top: CACTTCAGATAACTACACCGTTTT

CXCR4 target bottom: CGGTGTAGTTATCTGAAGTGCGGTG

### 2.2 Flow cytometry

CXCR4 and CXCR7 expression was detected with 5 μg/mL allophycocyanin (APC)-labeled rat anti-human CD184 (CXCR4) monoclonal antibody (clone 12G5: BioLegend) and 10 μg/mL phycoerythrin (PE)-labeled mouse anti-human CXCR7 monoclonal antibody (clone 8F11-M16: BioLegend), respectively. CCR7 expression was detected with biotin-labeled rat anti-human CCR7 monoclonal antibody (clone 3D12: BD Biosciences, 1:20 dilution) and 1 μg/mL Dylight 649-labeled streptavidin (BioLegend). Binding of soluble chemokine chimeras containing the human immunoglobulin constant region (CXCL12-Fc, CCL19-Fc, and control-Fc; gifts from Dr. K. Hieshima, Kindai University, Osaka, Japan [15]) to H9 cells was detected by staining with PE-conjugated goat anti-human IgG antibody (Jackson Immuno Research, Inc.). The data analysis and interpretation were carried out using a BD LSRFortessa system and the FlowJo software (BD Biosciences).

### 2.3 RT-PCR analysis

Total RNA was purified using the RNA Basic Kit, and cDNA was synthesized using the Scriptase Basic cDNA-Kit (NIPPON Genetics Co., Ltd.). PCR was performed with KOD-FX Neo (TOYOBO Co., Ltd.), oligonucleotide primers, and 0.2 μg template cDNA. The amplified DNA products of CXCR7 (627-bp), CXCR4 (367-bp), CCR7 (530-bp), and beta-actin (838-bp) were analyzed by agarose gel electrophoresis. The signal intensities were determined using ImageJ software (NIH). The primer sequences used are described below.

CXCR7 forward: 5′-ATGGATCTGCATCTCTTCGA-3′

CXCR7 reverse: 5′-CAGCCACTCCTTGATGCTGT-3′

CXCR4 forward: 5′-AATCTTCCTGCCCACCATCT-3′

CXCR4 reverse: 5′-GACGCCAACATAGACCACCT-3′

CCR7 forward: 5′-TCCTTCTCATCAGCAAGCTGTC-3′

CCR7 reverse: 5′-GAGGCAGCCCAGGTCCTTGAAG-3′

Beta-actin forward: 5′-ATCTGGCACCACACCTTCTACAATGAGCTGCG-3′

Beta-actin reverse: 5′-CGTCATACTCCTGCTTGCTGATCCACATCTGC-3′

### 2.4 Preparation of conditioned cell culture medium

The cells in ice-cold PBS containing 5 mM MgCl_2_ were allowed to stand for 30 min on ice. Cells were then resuspended at 2 × 10^6^ cells/mL with 100 ng/mL recombinant human CXCL12 (Sigma) or PBS containing 0.1% bovine serum albumin (BSA) and incubated for 30 min at 37 °C. After centrifugation, the supernatant was collected and filtered with a 0.45-μm pore size filter and, subsequently, with a 0.22-μm filter (Millex). The filtered medium was centrifuged for 20 min at 7500×*g* using Amicon Ultra-4 10 K centrifugal filter devices (Merck Millipore). The 20-fold concentrated conditioned medium (CM) of each treatment was used for the following experiments.

### 2.5 Transwell cell migration assay

CXCL12 or CCL19-dependent cell migration was performed with a transwell cell culture chamber with a membrane diameter of 6.5 mm and a pore size of 8-μm (Corning, NY, USA). The parental H9 and CXCR4-KO cells were fluorescently stained with 10 μM CFSE (Invitrogen) or 1 μM CytoRed (Dojindo, 1 μM), respectively, for 30 min at 37 °C. The stained parental H9 alone, CXCR4-KO alone, or their 1:1 mixture was added to the upper chamber at 1 × 10^6^ cells/well, and the chemokine solutions of 100 ng/mL recombinant human CCL19 (R & D Systems) or human CXCL12 (Sigma) in RPMI1640 containing 0.1% BSA were added to the lower layer. Alternatively, only CXCR4-KO cells were added to the upper chamber at 1 × 10^6^ cells/well, and the parental H9 cells at 1 × 10^6^ cells/well with CXCL12 were added to the lower chamber. After incubation for 2 h at 37 °C in a CO_2_ incubator, the number of cells that had migrated to the lower chamber were analyzed by flow cytometry.

### 2.6 Time-lapse video microscopic analysis

Time-lapse analysis was performed with a real-time cell migration analyzer (EZ-TAXIScan; GE healthcare bioscience). The 20-fold concentrated CM of CXCL12-treated cells (CXCL12 CM) or that of the control treatment (control CM) were added at a ratio of 1:10 to the cell suspension at 1 × 10^8^ cells/mL. Immediately after the addition of each CM, 1 μL of the cell suspension was loaded into each well of the microchamber, and 100 ng of recombinant human CCL21 (R & D Systems) was applied to the contra-wells. In some cases, 1 μL of 20-fold concentrated CXCL12 CM or control CM was applied to the contra-wells. The images of the migrating cells on cover glasses were automatically captured by a CCD camera for 2 h at 37 °C. The qualitative analyses of cell migration were performed using FIJI Image J software (NIH).

### 2.7 Ethics statement

The experimental protocols were approved by the Ethics Review Committee of Kindai University. All experiments were conducted in accordance with the approved guidelines from Kindai University.

## 3 Results

### 3.1 CXCL12-stimulated cells enhanced CXCR4-KO cell migration

**Fig. 1:**
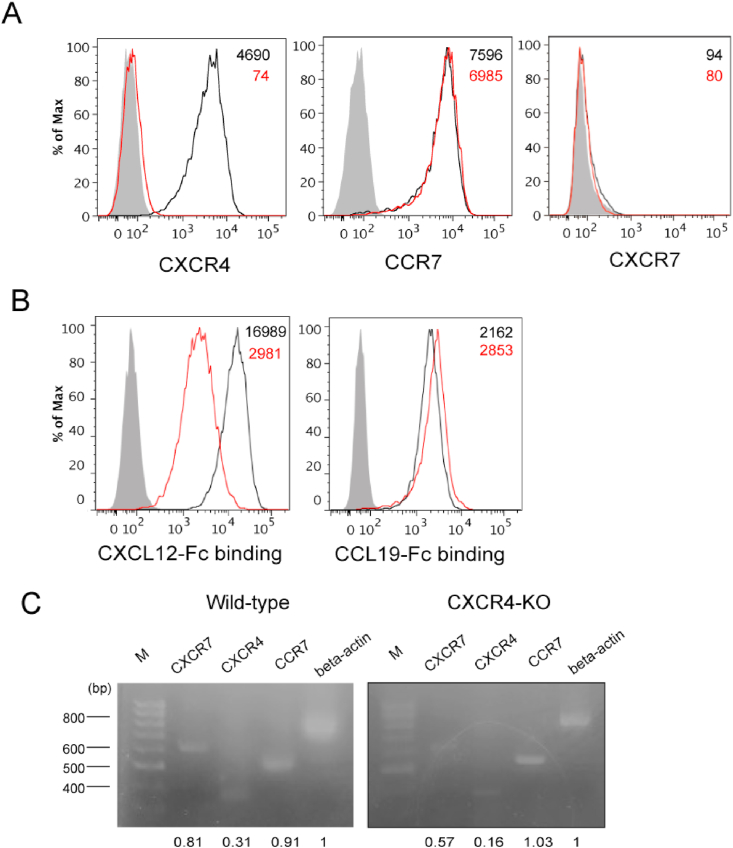
Expression of CXCR4, CCR7, CXCR7 and their ligand binding levels in CXCR4 knockout (KO) cells. (A) Wild-type cells (black line) and CXCR4-KO cells (red line) were stained with anti-CCR7, anti-CXCR4, anti-CXCR7, or isotype control antibodies (gray histogram) and analyzed by flow cytometry. The numerical values in the figure indicate the mean fluorescent intensity (MFI) of each histogram. (B) CCL19-Fc and CXCL12-Fc levels in Wild-type cells (black line) and CXCR4-KO cells (red line) were analyzed by flow cytometry. Gray histograms represent Control-Fc binding levels. The numbers in the figure indicate the MFI of each histogram. (C) The mRNA levels of CXCR4, CCR7, CXCR7 in Wild-type and CXCR4-KO cells were analyzed by RT-PCR. The gene expression levels relative to beta-actin are shown at the bottom. (For interpretation of the references to colour in this figure legend, the reader is referred to the Web version of this article.)

To assess the possibility that CXCL12-stimulated cells produce secondary chemoattractants that affect surrounding cell migration, we examined the effect of CXCL12-stimulated parental H9 cells (WT) on the surrounding CXCR4-KO cells. Under flow cytometric analysis, CXCR4 expression in CXCR4-KO cells was almost undetectable, whereas CCR7 expression was comparable between the WT and the CXCR4-KO cells (Fig. 1A). We confirmed that the cell surface expression of CXCR7 was undetectable in both CXCR4-KO and WT cells. We found that CXCL12-Fc binding was significantly reduced in CXCR4-KO cells compared to WT cells, suggesting the functional deficiency of CXCR4 in the CXCR4-KO cells (Fig. 1B). Conversely, CXCL12-Fc binding was moderately detected in CXCR4-KO cells, suggesting that CXCL12 binds to another receptor, rather than to CXCR4 or CXCR7, in H9 cells. Consistent with CCR7 expression in CXCR4-KO cells, the CCL19-Fc binding level in CXCR4-KO cells was comparable to that in the WT cells. Despite the lack of cell surface expression, modest CXCR7 signals were detected in both WT and CXCR4-KO cells by RT-PCR (Fig. 1C), suggesting that CXCR7 mRNA is expressed, but scarcely expressed on the cell surface as previously reported [16]. The CXCR4 mRNA expression in CXCR4-KO cells was decreased by about half compared to WT cells, indicating that CXCR4 expression was hampered at transcription and translation levels.

**Fig. 2:**
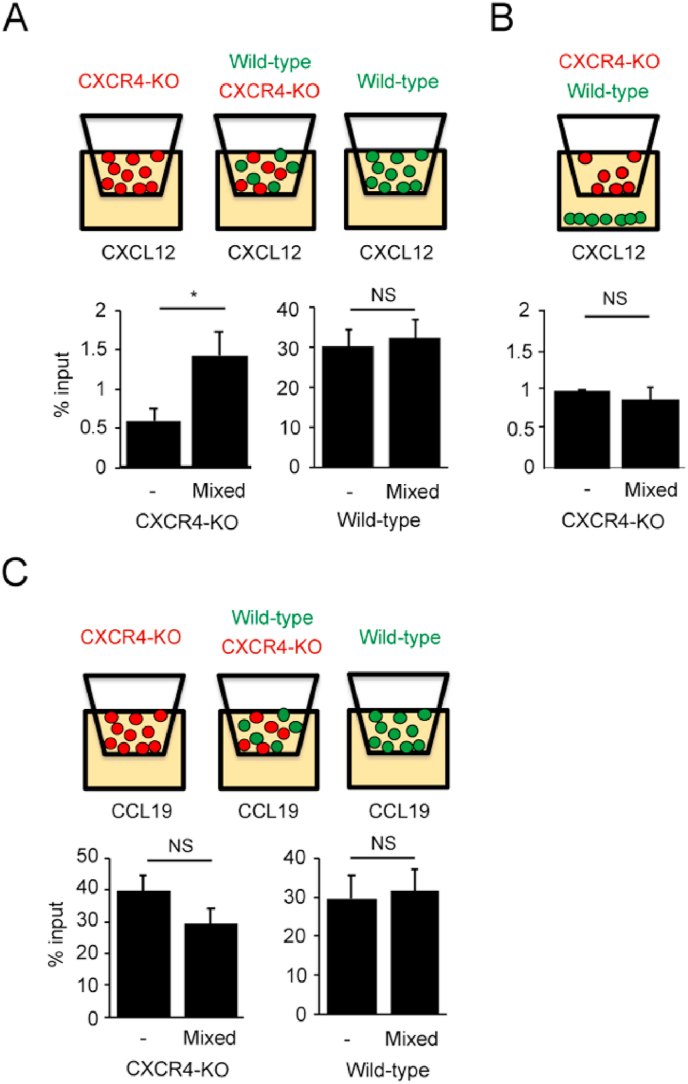
A schematic view of transwell migration assay and the cell migration ratio of WT and CXCR4-KO cells stained with different fluorescent dyes. The efficiencies of CXCL12-dependent cell migration were calculated when WT and CXCR4-KO cells were added to the upper wells (A) or WI cells were added to the lower layer (B). Each data shows the mean ± SEM obtained from five and three independent experiments for panel (A) and (B), respectively. All significance tests were performed by Student's t-test (*p < 0.05). NS, not significant. (C) The efficiencies of CCL19-dependent cell migration were calculated when WT and CXCR4-KO cells were added to the upper wells. Each data shows the mean ± SEM obtained from three independent experiments.

To examine the possibility that WT cells affect CXCR4-KO cell migration, we compared CXCR4-KO cell migration with or without WT cells. To this end, we stained WT cells and KO cells, respectively, with different fluorescent dyes and subjected them to CXCL12-dependent cell migration with either CXCR4-KO cells alone or with CXCR4-KO cells pre-mixed with WT cells (Fig. 2A). In the transwell cell migration assay, CXCR4-KO cell migration was significantly increased to approximately 2-fold when CXCR4-KO cells were mixed with WT cells. In contrast, the cell migration of WT cells to CXCL12 was not affected by the presence of KO cells. When CXCR4-KO cell migration was examined with or without WT cells in the lower layer chambers supplemented with CXCL12, no significant change in CXCR4-KO cell migration was observed, irrespective of the presence of WT cells (Fig. 2B). From the above results, we speculated that WT cells affect CXCR4-KO cell migration when they are in close proximity. In contrast, CCR7 ligand chemokine (CCL19)-dependent CXCR4-KO cell migration was not significantly affected by mixing with WT cells (Fig. 2C), suggesting that the WT cells stimulate CXCR4-KO cell migration, which is selectively increased in the presence of CXCL12.

### 3.2 CXCL12-processed cell culture medium affected CXCR4 KO cell migration

**Fig. 3:**
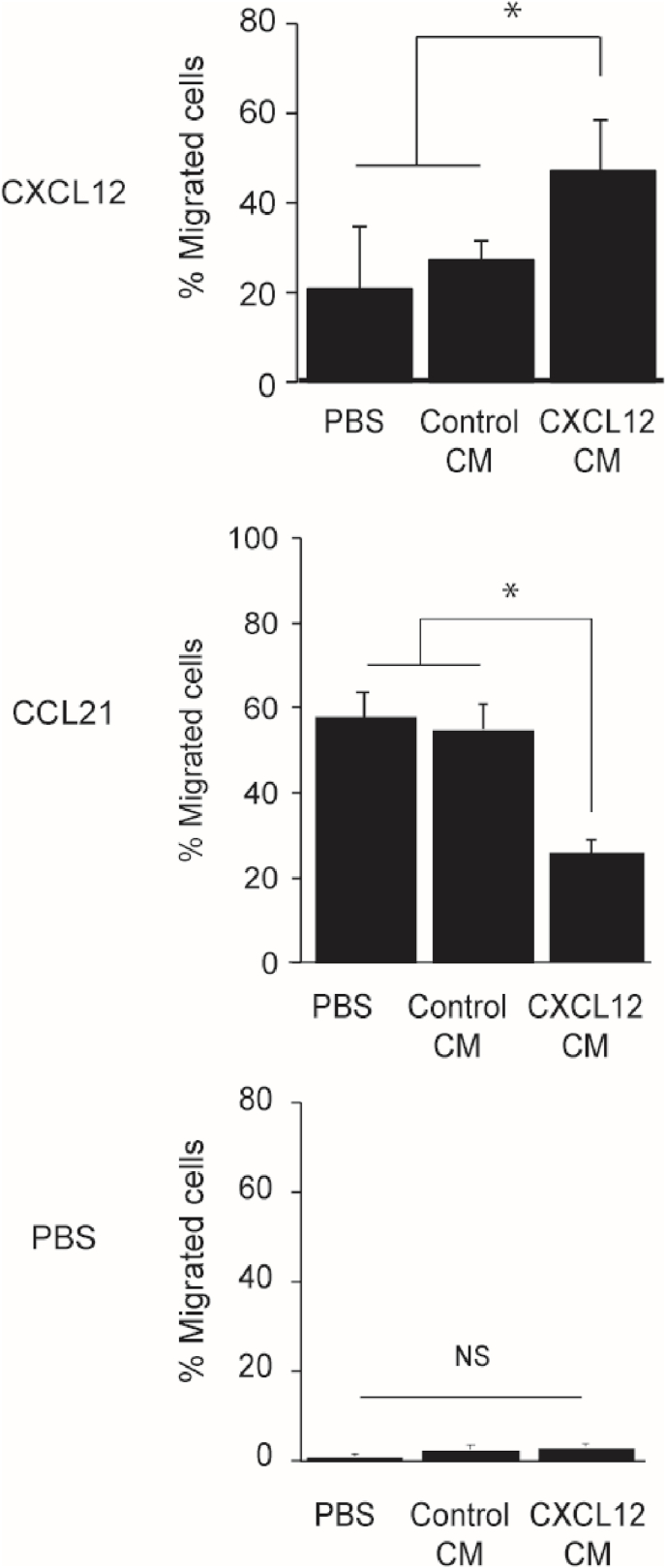
The effects of control-treated or CXCL12-treated CM of WT cells on CXCR4-KO cell migration to CXCL12, CCL21, or PBS. CXCR4-KO cells equivalent to 1 × 105 cells in 2-fold concentrated CMs were loaded into each well of the microchamber. The data show the mean ± SEM obtained from three independent experiments. Statistical analysis of the obtained data was performed by Tukey’ s test (*p < 0.05 against control).

To investigate the possibility that CXCL12-stimulated WT cells secrete paracrine factors that promote CXCR4-KO cell migration, we examined the effect of a CM from WT cells on CXCR4-KO cell migration. We prepared a concentrated cell culture media of CXCL12-treated WT cells (CXCL12 CM) and a control BSA-treated CM (control CM) by centrifugation with a molecular weight cut-off of 10 K. As shown in Fig. 3, CXCL12-dependent CXCR4-KO cell migration increased when the cells were pre-incubated with CXCL12 CM, but not with the control CM (Fig. 3). In contrast, the CXCL12 CM significantly suppressed CXCR4-KO cell migration in response to the CCR7 ligand, CCL21. These results suggest that CXCL12-treated WT cells secrete paracrine factors that promote CXCL12-dependent cell migration and suppress CCR7 ligand-dependent CXCR4-KO cell migration.

**Fig. 4:**
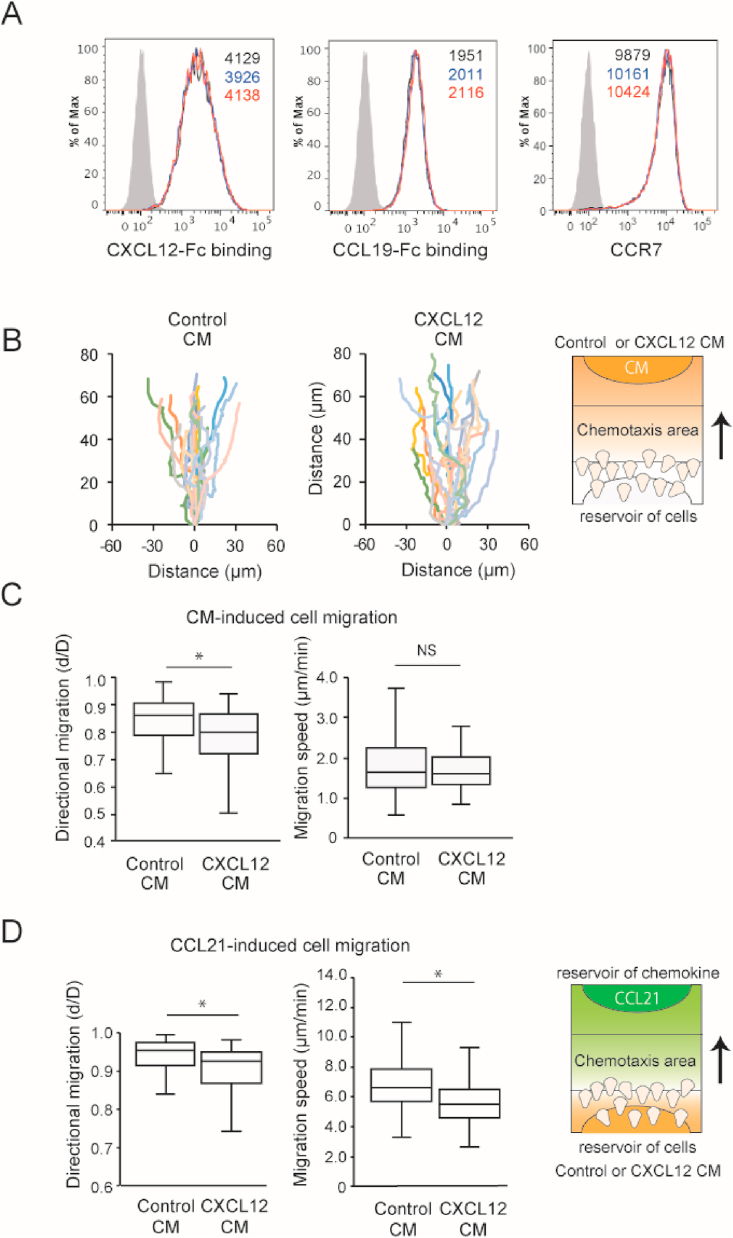
The effects of the control-treated or CXCL12-treated CM on the CXCR4-KO cell migration behavior. (A) The CXCL12-Fc binding. CCL19-Fc binding, and CCR7 expression levels in CXCR4-KO cells after treatment with PBS (black). control CM (blue), or CXCL12 CM (red) were analyzed by flow cytometry. Gray histograms represent the binding level of Control-Fc or an isotype control antibody. (B) The trajectory of migrated CXCR4-KO cells in the presence of CXCL12-treated or control-treated CM. The representative results from seven independent experiments are shown. Each line shows the trajectory of individual cell (control-treated CM. n = 20 and CXCL12-treated CM, n = 18). (C) The directional migration (left) and migration speed (right) of the CXCR4-KO cells in response to the control-processed or the CXCL12-processed CM. The directionality (d/D) was calculated by the ratio of the shortest distance until the cell reached the end point and the distance actually traveled. In the control-processed CM, 122 cells were analyzed, and 124 cells were analyzed in the CXCL12-processed CM. (D) The directional migration (left) and migration speed (right) of CCL21-induced CXCR4-KO cell migration in the presence of control-processed CM or CXCL12-processed CM. In the PBS-treated CM, 100 cells were analyzed. 95 cells were analyzed in the control-treated CM. and 80 cells were analyzed in the CXCL12-treated CM. The statistical difference was determined by two-tailed Mann-Whitney’ s *U* test and depicted with *p < 0.05. NS, not significant. (For interpretation of the references to colour in this figure legend, the reader is referred to the Web version of this article.)

To gain insight into the mechanism underlying the CXCL12 CM's role in cell migration, we examined whether the CXCL12 CM affected chemokine binding in CXCR4-KO cells. As shown in Fig. 4A, neither of the CMs affected the CCR7 expression, CXCL12-Fc binding, or CCL19-Fc binding, suggesting that the enhancing and suppressive effects on CXCR4 and CCR7-dependent cell migration, respectively, were not caused by changes in ligand–receptor binding.

Next, we examined the role of CXCL12 CM in migratory behavior (directionality, migration speed). The analysis revealed that the CXCL12 CM decreased the directionality (the ratio of the shortest distance from the origin to the end point of migration to the distance actually migrated) in CXCR4-KO cell migration compared to the control CM (Fig. 4B), but did not significantly affect the migration speed (Fig. 4C). These results suggest that CXCL12 CM contains substances that change cell migration behavior. When CCL21 was used as a chemoattractant, both the directionality and the speed of cell migration was decreased in the CXCL12 CM (Fig. 4D), in agreement with the suppressive role of CXCL12 CM on CCR7 ligand-dependent cell migration (Fig. 3B).

## 4 Discussion

### 4.1 Secondary factors produced by T lymphocytes after CXCL12 stimulation

In this study, we found that the conditioned medium of CXCL12-treated cells promoted CXCR4-deficient cell chemotaxis, and the CXCL12 CM showed a suppressive effect on CCR7 ligand-dependent cell motility by affecting the directionality and speed of chemotaxis. These results support the idea that the CXCL12/CXCR4 axis causes the production of a signal-relay molecule that contributes to chemokine-dependent lymphocyte migration, similar to that reported in neutrophils [13].

We found that the CXCL12 CM promoted CXCR4-KO cell migration in the presence of CXCL12, suggesting that CXCR4-KO cells responded to CXCL12 via a CXCL12-binding receptor other than CXCR4. CXCR7 is known as a CXCL12 receptor with approximately 10 times higher affinity for CXCL12 than for CXCR4 [17,18] and mediates cell migration in certain T lymphocyte cell lines [19]. Numerous studies using cancer and primary cells have shown that CXCR7 is a functional receptor in various cell types [20,21], although each contribution of CXCR7 or CXCR4 to CXCL12-mediated cell migration is still controversial. Some studies have demonstrated that CXCR7 does not conjugate with G proteins, and it acts as a scavenger receptor that sequesters CXCL12 to control the CXCL12 gradient [22,23], whereas others have shown that it activates signaling pathways including arrestin recruitment [21]. In T lymphocytes, CXCR7 by itself does not activate G proteins, but it supports CXCR4 signaling through formation of CXCR4/CXCR7 complexes [24]. In HuT78-derived H9 cells used in this study, the cell surface CXCR7 expression was undetectable, as previously reported in HuT78 cells [25]. Therefore, the contribution of the CXCL12/CXCR7 axis to CXCR4-KO cell migration is considerably low. As a CXCL12 binding molecule other that CXCR7, a proteoglycan syndecan-4 is known to directly binds to CXCL12 and triggers cell activation signals [26]. Since syndecan-4 is expressed in HuT78 cells [27], it potentially contributes to CXCL12-Fc binding and mediates CXCL12-dependent cell migration in the CXCR4-KO cells.

We have not yet successfully identified the secondary factors produced by the CXCL12-treated lymphocytes. It has been reported that HuT78 cells efficiently migrate to IP-10/CXCL10, BCA 1/CXCL13, SLC/CCL21 and CTACK/CCL27 [28]. Our preliminary qPCR analysis of chemokine expression levels showed no significant changes in CCL19 and CCL27 after CXCL12 stimulation, and reduction of CCL21 and CXCL10 by nearly half. These results suggest that the above-mentioned chemokines are unlikely to be the secondary stimulants in the CXCL12 CM. Alternatively, substances with non-directional chemokinetic action on T lymphocytes, such as lysophosphatidic acid (LPA) or interferon-α2, may be involved in the phenomenon [29,30]. LPA enhances CXCL12-dependent chemotaxis and motility in T cells and has a dual action by promoting motility and directional cell migration or enhancing non-directed cell migration and inducing chemorepulsion in the presence of CCL21 [[30], [31], [32], [33]]. On the other hand, we cannot exclude the possibility that the remaining CXCL12 in the concentrated CM caused CXCR4-KO cell migration, although the CM was prepared using an ultrafiltration membrane with a molecular weight cut-off of 10 K. Another possibility is that the remaining CXCL12 in the concentrated CM interacted with CXCL12 binding molecules such as syndecan-4, and then activated intracellular signaling in CXCR4-KO cells. Additional studies into the substances secreted by lymphocytes after CXCL12 stimulation will lead to the identification of the molecules which control the surrounding cell motility.

### 4.2 Biological significance of T lymphocyte-derived paracrine factors by CXCL12 stimulation

In our previous study, we demonstrated that the activation of CXCR4 signaling promotes CCR7 ligand-dependent *in vitro* T cell chemotaxis and *in vivo* cell trafficking to lymph nodes, suggesting that multiple chemoattractants function cooperatively to induce efficient leukocyte recruitment to target tissues [7,34]. In those studies, we demonstrated that CXCR4-mediated signaling induces CCR7 homo-dimerization and facilitates CCR7 ligand binding on the cell surface, and subsequently enhances the reactivity of the cells to CCR7 ligands [34,35]. Conversely, we found in this study that the paracrine factor induces an inhibitory signaling on CCR7-dependent chemotaxis in the absence of CXCR4-mediated signaling. According to these findings, we speculate that the paracrine factor may contribute to the maintenance of lymphocyte responsiveness to the first chemokine in settings of limited ligand availability *in vivo*, and prevent them from responding to other chemokines*.* Further investigation is needed to clarify whether the secondary paracrine factors contribute to propagate a chemokine signal to the surrounding cells, as has been demonstrated in neutrophils.

## 5 Conclusions

We revealed that CXCL12 contributes to lymphocyte navigation in concert with a secondary paracrine factor. Our results shed new light on the mechanisms for lymphocyte migration, particularly how chemokine-stimulated cells propagate cell migration signals to the surrounding cells.

## Author contributions

K.K., M.E., and H.H. designed the experiments; K.K., M.E., and D.K. performed most of the experiments and analysis; H.H. wrote the manuscript. All authors reviewed and approved the final manuscript.

## Declaration of competing interest

The authors declare that they have no known competing financial interests or personal relationships that could have appeared to influence the work reported in this paper.
